# Respiratory Syncytial Virus Infects Primary Neonatal and Adult Natural Killer Cells and Affects Their Antiviral Effector Function

**DOI:** 10.1093/infdis/jiy566

**Published:** 2018-09-25

**Authors:** Elisabeth A van Erp, Dorien Feyaerts, Maxime Duijst, H Lie Mulder, Oliver Wicht, Willem Luytjes, Gerben Ferwerda, Puck B van Kasteren

**Affiliations:** 1 Centre for Infectious Disease Control, National Institute for Public Health and the Environment, Bilthoven; 2 Section of Pediatric Infectious Diseases, Laboratory of Medical Immunology, Radboud Institute for Molecular Life Sciences, Nijmegen, The Netherlands; 3 Radboud Center for Infectious Diseases, Radboudumc, Nijmegen, The Netherlands

**Keywords:** RSV, NK cells, interferon-γ, antibody, ADE

## Abstract

**Background:**

Respiratory syncytial virus (RSV) is a major cause of severe acute lower respiratory tract infections in infants. Natural killer (NK) cells are important antiviral effector cells that likely encounter RSV in the presence of virus-specific (maternal) antibodies. As NK cells potentially contribute to immunopathology, we investigated whether RSV affects their antiviral effector functions.

**Methods:**

We assessed the phenotype and functionality of primary neonatal and adult NK cells by flow cytometry after stimulation with RSV or RSV-antibody complexes.

**Results:**

We demonstrate for the first time that RSV infects neonatal and adult NK cells in vitro. Preincubation of virus with subneutralizing concentrations of RSV-specific antibodies significantly increased the percentage of infected NK cells. Upon infection, NK cells were significantly more prone to produce interferon-γ, while secretion of the cytotoxicity molecule perforin was not enhanced.

**Conclusions:**

Our findings suggest that (antibody-enhanced) RSV infection of NK cells induces a proinflammatory rather than a cytotoxic response, which may contribute to immunopathology. Considering that most RSV vaccines currently being developed aim at inducing (maternal) antibodies, these results highlight the importance of understanding the interactions between innate effector cells and virus-specific antibodies.

## Teaser

Respiratory syncytial virus (RSV) can infect neonatal and adult natural killer cells, thereby inducing a proinflammatory rather than a cytotoxic response. In subneutralizing concentrations, virus-specific antibodies can enhance infection. These findings provide novel insights into the potential mechanisms underlying severe RSV immunopathology.

Human respiratory syncytial virus (RSV) is a major cause of severe lower respiratory tract disease in infants [1]. There are currently no market-approved vaccines or antivirals available against this virus. The estimated global burden of RSV-associated severe acute lower respiratory infection was 33.1 million in 2015, with an estimated 118200 deaths in children <5 years of age [2]. Hospitalization for severe RSV-mediated disease peaks between 6 weeks and 6 months of life [3], when infants mainly depend on maternal antibodies and their innate immune system for protection against infectious diseases. Despite extensive research efforts, the immunological determinants of severe RSV-mediated disease remain elusive.

Natural killer (NK) cells are innate lymphocytes that play an important role in the control of viral lung infections. Within days after infection, large numbers of NK cells are recruited to the lung and become activated [4, 5]. NK cells have multiple mechanisms to combat viral replication: (1) death receptor–mediated cytolysis to kill virus-infected target cells, (2) production of proinflammatory cytokines with antiviral activity (eg, interferon gamma [IFN-γ]), and (3) antibody-dependent cell-mediated cytotoxicity, in which NK cells bind antibody-coated virus-infected target cells via Fc gamma receptor III (FcγRIII)/CD16 followed by target cell lysis.

The role of NK cells during RSV-induced disease is still unclear. In mice, increased numbers of NK cells are present in the lungs early after RSV infection [4–6]. In this model, the presence of NK cells is sufficient to eliminate RSV infection [7] and depletion of NK cells significantly increases viral loads [8]. However, increasing evidence suggests that NK cells also contribute to inflammatory lung injury, for example via the production of IFN-γ [5, 8, 9].

 There are contradictory reports on NK cells in humans during severe RSV infection. In infants, the proportion of NK cells has been reported both to be decreased [10–13] or increased [14, 15] in comparison with healthy controls or infants with mild symptoms. NK cell gene expression in whole blood was reported to be downregulated in infants with severe RSV disease compared with controls [16]. Therefore, definitive conclusions about the role of NK cells in RSV infection and disease cannot be drawn from the data currently available.

In this study, we investigated whether interaction of RSV or RSV-antibody complexes with NK cells affects their function. Interestingly, we found that RSV infects NK cells and that infection influenced the effector function of both neonatal and adult NK cells. RSV-infected NK cells were more prone to produce IFN-γ than uninfected cells, while the percentage of perforin-secreting cells was not increased. We show that preincubation of RSV with subneutralizing concentrations of virus-specific antibodies increases the number of infected and, hence, IFN-γ–secreting NK cells. We propose that (antibody-enhanced) infection of NK cells with RSV may contribute to immunopathology, through induction of a proinflammatory rather than a cytotoxic response in these cells.

## MATERIALS AND METHODS

### Cells and Viruses

Peripheral blood mononuclear cells (PBMCs) were obtained from healthy volunteers at the National Institute for Public Health and the Environment (RIVM, the Netherlands). Cord blood mononuclear cells (CBMCs) from umbilical cords of healthy neonates born by cesarean delivery were collected at Radboudumc Nijmegen (the Netherlands). Blood was collected in heparin tubes and the mononuclear fraction was isolated by density gradient centrifugation (Lymphoprep, Nycomed). NK cells were purified by negative selection using a CD56^+^ NK cell isolation kit (Miltenyi Biotec). In all experiments, NK cells were gated as the CD3(^–^), CD56(^+^) population. Isolated cells were cultured in Iscove’s Modified Dulbecco’s Media (IMDM; Gibco) supplemented with 10% heat-inactivated fetal calf serum (hiFCS), 1% penicillin/streptomycin/glutamine (PSG, Gibco), and 5 ng/mL recombinant interleukin 15 (IL-15; Biolegend). CBMCs were stored at –135°C and thawed before NK cell isolation.

Vero cells (ATCC-CCL81) were propagated in Dulbecco’s modified Eagle’s medium supplemented with 5% hiFCS and 1% PSG. Human chronic myelogenous leukemic K562 cells (a kind gift from Jeannette Cany, Radboudumc Nijmegen) were propagated in IMDM, supplemented with 10% hiFCS and 1% PSG.

Recombinant RSV-X and RSV-X-GFP7 (encoding green fluorescent protein [GFP]) were propagated in Vero cells as described before [17, 18]. Virus stocks were purified between layers of 10% and 50% sucrose by ultracentrifugation. The 50% tissue culture infectious dose (TCID_50_)/mL was determined on Vero cells using the Spearman and Karber method [19].

### Plaque Reduction Neutralization Test

Using RSV-X-GFP7, a Vero-based plaque reduction neutralization test was performed for intravenous immunoglobulin (IVIg; KIOVIG, Baxalta), palivizumab (SYNAGIS, AbbVie), and the World Health Organization (WHO) International Standard for antiserum to RSV (National Institute for Biological Standards and Control [NIBSC]), as described previously [18]. Based on the neutralization curves, the amount of international units (IU) of anti-RSV neutralizing antibodies per milliliter was calculated for IVIg and palivizumab using the WHO standard as reference serum (containing 2000 IU/mL).

### RSV Infection

NK cells were infected with RSV-X-GFP7 or RSV-X by spinoculation for 1 hour at 700*g* at 20°C, followed by incubation for 1 hour at 37°C. A multiplicity of infection (MOI) of 1 based on titration on Vero cells was used. Next, cells were washed with phosphate-buffered saline and replenished with culture medium. For antibody-dependent enhancement (ADE) assays, RSV was preincubated with the indicated concentrations of IVIg or palivizumab for 10 minutes at 37°C, before spinoculation of NK cells. Incubation at 37°C was followed by flow cytometric analysis at the indicated time points using an LSR Fortessa X20 (BD Biosciences). RSV infection was blocked by coincubation with 100 nM fusion inhibitor (TMC-353121, MCE) [20]. FcγRIII/CD16 was blocked by preincubation of NK cells with 50 µg/mL anti-CD16 Fab fragments (3G8, Ancell). Infection was measured by GFP expression for RSV-X-GFP7 or with a fluorescein isothiocyanate (FITC)–conjugated RSV-G antibody (131-2G, Millipore). Productivity of NK cell infection was assessed by TCID_50_ of the cleared supernatants on Vero cells as described above.

### Flow Cytometric Phenotypic Characterization

The following fluorochrome-conjugated monoclonal antibodies were used to phenotypically characterize (RSV-infected) NK cells: CD3-APCAF750 (UCHT1, Beckman Coulter), CD16-PacificOrange (3G8, Thermo Fisher), CD56-ECD (N901, Beckman Coulter), CD85j-PerCP-Cy5.5 (ILT2, LILRB1; GHI/75, BioLegend), CD161-APC (191B8, Miltenyi), CD158a-AF700 (KIR2DL1; 143211, R&D Systems), CD158a/h-PC5.5 (KIR2DL1/S1; EB6B, Beckman Coulter), CD158b1/b2,j-PC7 (KIR2DL2/L3/S2; GL183, Beckman Coulter), CD158e1-BV421 (KIR3DL1; DX9, BioLegend), CD159a-APC (NKG2A; Z199, Beckman Coulter), CD159c-PE (NKG2C; 134591, R&D Systems), CD244-AF700 (2B4; C1.7, BioLegend), CD314-APC (NKG2D; ON72, Beckman Coulter), CD335-PC7 (NKp46; BAB281, Beckman Coulter), CD336-PE (NKp44; Z231, Beckman Coulter), CD337-PerCP-Cy5.5 (NKp30; P30-15, BioLegend), RSV-G-FITC (131-2G, Millipore). Cells were measured using a Navios flow cytometer (Beckman Coulter).

### NK Activation Assay

At 20 hours postinfection with RSV or RSV-antibody complexes, the NK cells were incubated for 4 hours in the absence or presence of K562 target cells together with brefeldin A (BD Bioscience) and CD107a-PE/Cy7 antibody (H4A3, Biolegend). Subsequently, cells were stained using the following antibodies: CD56-PE (HCD56, Biolegend), CD3-PerCP (SK7, BD Biosciences), RSV-G-FITC (131-2G, Millipore), IFN-γ–APC antibody (B27, BD Bioscience), perforin-BV421 (B-D48, Biolegend), and fixable viability dye eFluor780 (eBioscience).

### Statistical Analysis

Comparison of 2 groups or data points was performed by using a nonparametric Wilcoxon signed-rank test. Multiple comparisons were analyzed by using a nonparametric Friedman test, followed by Dunn multiple comparisons test. *P* values <.05 were considered statistically significant. All statistical analyses were performed with Prism 7 software (GraphPad).

### Ethics Statement

All blood donors (PBMCs) and mothers (CBMCs) provided written informed consent.

## RESULTS

### RSV Infects and Replicates in Primary Adult NK Cells

**Figure 1.**
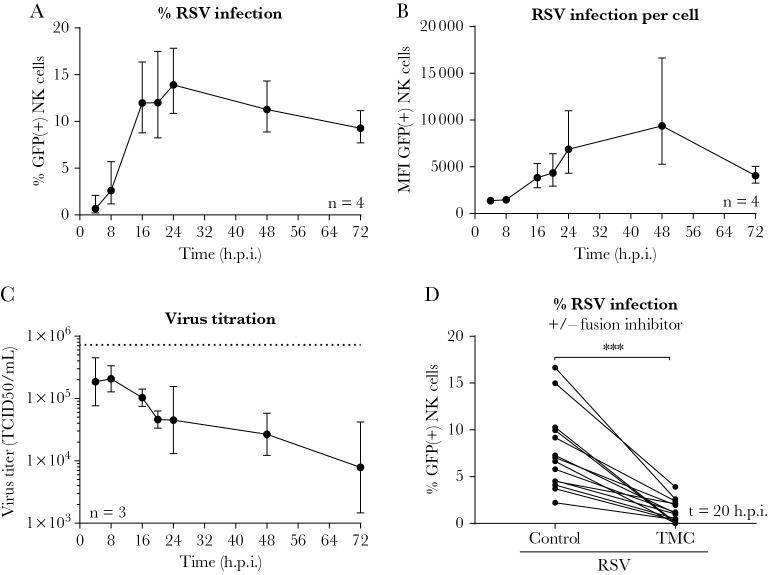
Respiratory syncytial virus (RSV) infects primary natural killer (NK) cells in vitro. *A* and *B*, Flow cytometric analysis showing the percentage (*A*) or median fluorescence intensity (*B*) of green fluorescent protein–positive cells for adult primary NK cells after spinoculation with RSV-X-GFP7 (multiplicity of infection = 1). Graphs depict geometric mean and standard deviation (SD) of 4 donors pooled from 2 independent experiments. *C*, The 50% tissue culture infectious dose of cleared RSV-infected NK cell supernatant on Vero cells. Graph depicts geometric mean and SD of 3 donors from 1 experiment. The dashed line depicts the retitration of the initial RSV inoculum. *D*, NK cells were spinoculated with RSV-X-GFP7 in the absence or presence of a fusion inhibitor (TMC) and infection was measured by flow cytometry at 20 hours postinfection. Data were pooled from 6 independent experiments and each set of paired data points represents an individual donor (n = 14). Wilcoxon signed-rank test was used for comparison between conditions (***P < .001). Abbreviations: GFP, green fluorescent protein; h.p.i., hours postinfection; MFI, median fluorescence intensity; NK, natural killer; RSV, respiratory syncytial virus; TCID_50_, 50% tissue culture infectious dose.

To assess the interaction of RSV with NK cells, primary adult NK cells (>95% CD3[^–^] cells) were spinoculated with RSV-X-GFP7 at a Vero-based MOI of 1. We observed steadily increasing expression of virus-encoded GFP, which is indicative of viral replication. In a time-course experiment, the maximum percentage of GFP-positive NK cells (CD3[^–^], CD56[^+^]) was observed at 24 hours postinfection (Figure 1A). The level of RSV infection showed considerable donor variability, and reached a maximum of up to 20% infected NK cells in some donors. The amount of intracellular GFP increased over time as shown by the Median Fluorescence Intensity (MFI) (Figure 1B). TCID_50_ assays of the NK cell supernatant showed a decrease in viral titer over time, suggesting that little or no infectious viral particles were released (Figure 1C). Inoculation of NK cells with RSV-X-GFP7 in the presence of a fusion inhibitor (TMC) showed efficient inhibition of NK cell infection (Figure 1D), indicating that viral entry was required for GFP detection and depended on the fusion (F) protein. The TMC vehicle control (dimethyl sulfoxide) showed no effect on infection (Supplementary Figure 1*A*). Moreover, increasing the titer of the inoculum resulted in considerably higher infection rates (Supplementary Figure 1*B*), suggesting that RSV does not exclusively infect a minor NK cell subpopulation. Altogether, these data show that RSV infects primary adult NK cells in vitro.

### Nonneutralizing RSV-Specific Antibodies Enhance NK Cell Infection

**Figure 2.**
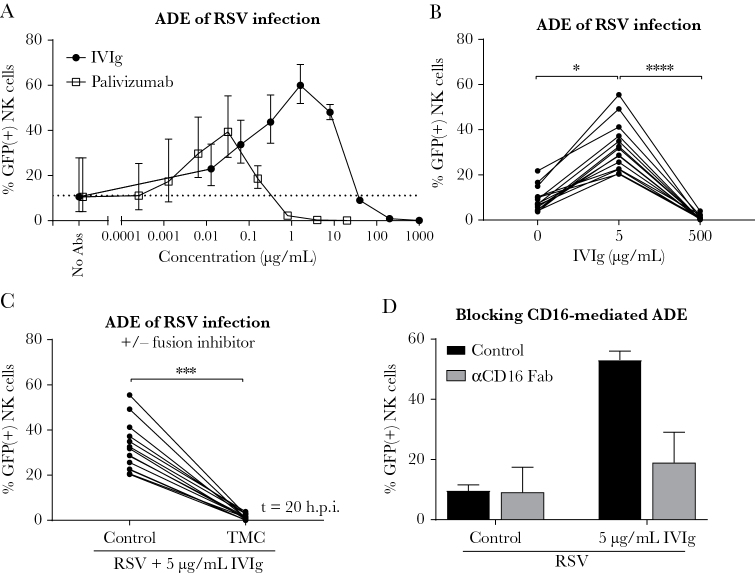
Antibody-dependent enhancement of respiratory syncytial virus (RSV) infection in natural killer (NK) cells. *A*, Infection of adult primary NK cells in the presence of RSV-X-GFP7-antibody complexes (with the indicated concentration of intravenous immunoglobulin [IVIg] or palivizumab). Graph depicts the geometric mean and standard deviation (SD) of 3 donors from 1 experiment. Dashed line depicts the geometric mean percentage green fluorescent protein (GFP)–positive cells in absence of antibodies (no antibodies). *B*, Infection of NK cells with RSV-X-GFP7 or (sub)neutralizing RSV-antibody complexes (5 µg/mL or 500 µg/mL IVIg). Data were pooled from 6 independent experiments and each set of paired data points represents an individual donor (n = 14). *C*, Infection of NK cells with subneutralizing RSV-antibody complexes (5 µg/mL IVIg) in the absence or presence of a fusion inhibitor (TMC). Data were pooled from 6 independent experiments and each set of paired data points represents an individual donor (n = 14). *D*, Infection of NK cells in the absence or presence of subneutralizing RSV-antibody complexes (5 µg/mL IVIg) with 50 µg/mL CD16-blocking Fab fragments. Graph depicts the geometric mean and SD of 3 donors from 1 experiment. All GFP measurements were performed by flow cytometry at 20 hours postinfection. Nonparametric Friedman test with Dunn multiple comparisons test was used for comparisons between multiple conditions (*P < .05, ****P < .0001). Wilcoxon signed-rank test was used for comparison between 2 conditions (***P < .001). Abbreviations: Abs, antibodies; ADE, antibody-dependent enhancement; GFP, green fluorescent protein; h.p.i., hours postinfection; IVIg, intravenous immunoglobulin; NK, natural killer; RSV, respiratory syncytial virus.

The vast majority of individuals infected with RSV possess RSV-specific antibodies, which vary in concentration and are either maternally derived through transplacental transfer or induced by previous exposure. NK cells that are recruited to the lung most likely encounter viral particles in complex with these antibodies. Since immunoglobulin G levels in the lung are lower than those found in serum [21], even neutralizing concentrations of serum antibodies may be accompanied by nonneutralizing antibody levels in the lung. Here we show that, while high antibody concentrations result in virus neutralization, incubation of NK cells with RSV-antibody complexes formed at subneutralizing concentrations results in ADE of infection (Figure 2A). This was shown for both IVIg, naturally containing RSV-specific antibodies, and palivizumab, a RSV-specific monoclonal antibody targeting the F protein. IVIg showed maximum enhancement around 1 μg/mL, corresponding to 0.0027 IU/mL of anti-RSV neutralizing antibodies. For palivizumab, maximum enhancement was observed at 0.03 μg/mL, corresponding to 0.0013 IU/mL. The standardized neutralizing antibody concentrations of IVIg and palivizumab were calculated using the WHO International Standard for antiserum to RSV as a reference (Supplementary Figure 1*C*).

Incubation of NK cells with RSV-antibody complexes formed at subneutralizing antibody concentrations (5 µg/mL or 0.014 IU/mL IVIg), resulted in up to 4-fold increased infection compared to the absence of antibodies (Figure 2B). Neutralizing antibody concentrations (500 µg/mL or 1.4 IU/mL IVIg) completely inhibited infection. ADE of infection was completely blocked in the presence of TMC, indicating that viral entry still depended on the F protein (Figure 2C). ADE of NK cell infection seems to involve CD16/FcγRIII, as incubation with CD16-blocking Fab fragments decreased infection in the presence but not in the absence of RSV-specific antibodies, although this difference was not statistically significant (Figure 2D). Spinoculation was used to enhance infection in all experiments, but this had no effect on the fold increase of antibody-enhanced infection compared to regular infection (Supplementary Figure 1*D*).

### Phenotypic Characterization of RSV-Infected NK Cells

**Figure 3.**
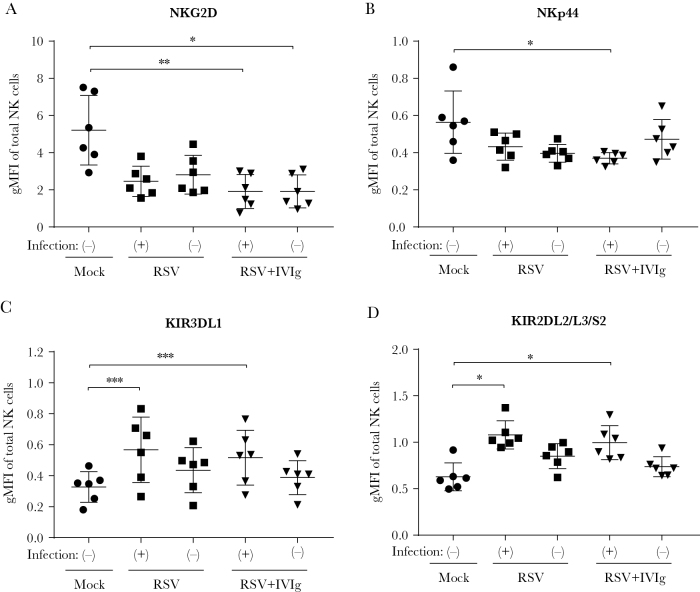
Phenotypic characterization of respiratory syncytial virus (RSV)–infected natural killer (NK) cells. *A–C*, Adult primary NK cells were inoculated with RSV-X or subneutralizing RSV-X antibody complexes (5 µg/mL intravenous immunoglobulin) and stained with 3 different antibody panels for flow cytometric analysis at 20 hours postinfection. RSV-infected and uninfected populations within 1 well are indicated with (+) and (-), respectively. Geometric mean of fluorescence intensity is depicted for the markers with the most pronounced differences compared to mock-infected NK cells: activating receptors NKG2D (*A*) and NKp44 (*B*), and KIRs KIR3DL1 (*C*), which is inhibitory, and KIR2DL2/L3/S2 (*D*), which can be either activating or inhibiting. Graphs depict the geometric mean and standard deviation of 6 donors pooled from 2 independent experiments. Nonparametric Friedman test with Dunn multiple comparisons test was used for comparison between multiple conditions (*P < .05, **P < .01, ***P < .001). Abbreviations: gMFI, geometric mean fluorescence intensity; IVIg, intravenous immunoglobulin; KIR, killer cell immunoglobulin-like receptor; NK, natural killer; RSV, respiratory syncytial virus.

Next, we set out to determine the phenotypic characteristics of RSV-infected NK cells using an extensive NK cell receptor panel. Those receptors showing the most pronounced effect upon infection are shown in Figure 3; the remaining receptors are depicted in Supplementary Figure 2. In the presence of subneutralizing RSV-antibody complexes, the activation markers NKG2D and NKp44 are downregulated (Figure 3A and 3B). The presence of RSV alone has a similar, but nonsignificant effect. In contrast, the HLA-C–specific killer cell immunoglobulin-like receptors KIR3DL1, which is inhibitory, and KIR2DL2/L3/S2, which can be activating or inhibiting, were more abundantly expressed on RSV-infected NK cells compared to mock-infected cells (Figure 3C and 3D). Taken together, although the effect on individual markers is not very dramatic, RSV-infected NK cells appear to be skewed toward an inhibitory phenotype.

### Neonatal NK Cells Are Susceptible to (Antibody-Enhanced) RSV Infection

**Figure 4.**
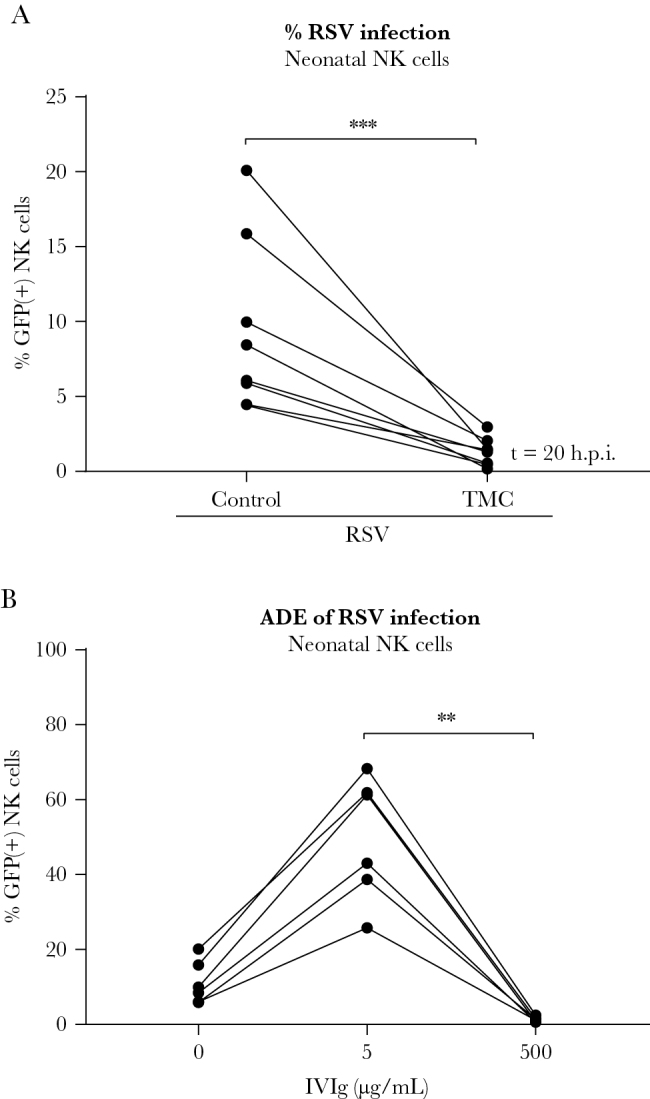
Neonatal natural killer (NK) cells are susceptible to respiratory syncytial virus (RSV) infection. Human neonatal NK cells were spinoculated with RSV-X-GFP7 (multiplicity of infection = 1). *A*, Percentage infected cells in absence or presence of a fusion inhibitor (TMC) as measured by flow cytometry. Data were pooled from 2 independent experiments and each set of paired data points represents an individual donor (n = 8). *B*, Percentage of infected cells after inoculation with RSV or RSV-antibody complexes (5 µg/mL or 500 µg/mL intravenous immunoglobulin). Data were pooled from 2 independent experiments and each set of paired data points represents an individual donor (n = 6). Wilcoxon signed-rank test was used for comparison between 2 conditions (***P < .001). Nonparametric Friedman test with Dunn multiple comparisons test was used for comparison between multiple conditions (**P < .01). Abbreviations: ADE, antibody-dependent enhancement; GFP, green fluorescent protein; IVIg, intravenous immunoglobulin; NK, natural killer; RSV, respiratory syncytial virus.

RSV is known to cause the most severe symptoms in the first months of life [3]. Therefore, the cells that are present at the moment of severe RSV disease in infants may more closely resemble umbilical cord blood NK cells than adult cells. We found that also neonatal NK cells (>90% CD3[^–^] cells) could be infected by RSV in vitro, and that infection was blocked by the fusion inhibitor TMC (Figure 4A). Neonatal NK cells showed a 6-fold increase in infection upon incubation with RSV-antibody complexes formed at subneutralizing antibody concentrations (5 µg/mL IVIg) compared to the no-antibody control (Figure 4B). Neutralizing antibody concentrations (500 µg/mL IVIg) completely inhibited infection. Overall, these results show that neonatal NK cells are comparable to adult NK cells in their susceptibility to RSV infection.

### RSV Infection of NK Cells Induces IFN-γ Production

**Figure 5.**
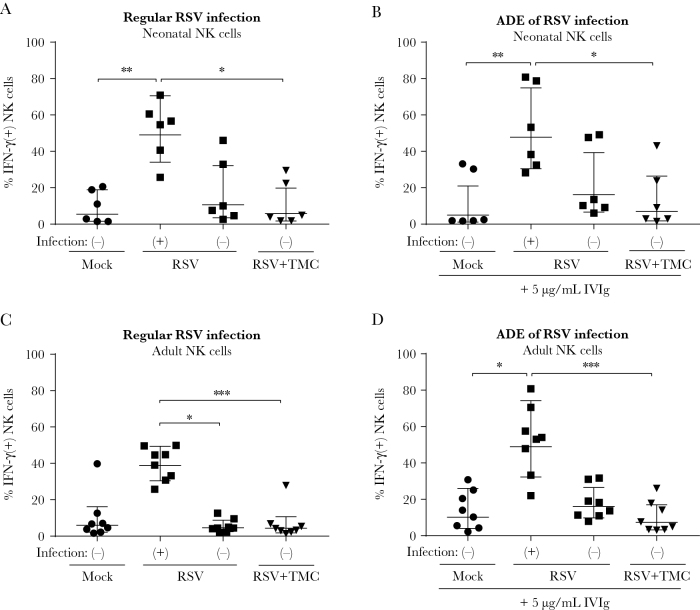
Respiratory syncytial virus (RSV) infection of natural killer (NK) cells induces interferon gamma (IFN-γ) production. Neonatal (*A* and *B*) or adult (*C* and *D*) NK cells were infected with RSV-X, inoculated with RSV-X and TMC, or mock-infected. RSV-infected and uninfected populations are indicated with (+) and (-), respectively. At 20 hours postinfection, NK cells were incubated for 4 hours with brefeldin A and subsequently stained for intracellular IFN-γ. *A* and *C*, Percentage of NK cells positive for intracellular IFN-γ is depicted for control NK cells, RSV(+) and RSV(-) cells within 1 RSV-inoculated well and for NK cells inoculated with RSV and TMC. *B* and *D*, Same as in *A* and *C* except for preincubation of the viral inoculum with 5 µg/mL intravenous immunoglobulin, resulting in antibody-dependent enhancement. All graphs depict geometric mean and standard deviation of 6 (neonatal) or 8 (adult) donors pooled from 2 (neonatal) or 4 (adult) independent experiments. Nonparametric Friedman test with Dunn multiple comparisons test was used for comparison between conditions (*P < .05, **P < .01, ***P < .001). Abbreviations: ADE, antibody-dependent enhancement; h.p.i., hours postinfection; IFN, interferon; IVIg, intravenous immunoglobulin; NK, natural killer; RSV, respiratory syncytial virus.

Because RSV infection of immune cells can have a profound effect on their functionality [22–24], we set out to explore the effect of RSV infection on NK cell functionality. First, we assessed IFN-γ production in 4 different NK cell populations: (1) mock-infected control cells; RSV-exposed (2) infected and (3) uninfected cells; and (4) uninfected cells exposed to RSV in the presence of TMC. The gating strategy for the NK cell activation assays is depicted in Supplementary Figure 3. RSV-infected neonatal NK cells showed significantly more IFN-γ–expressing cells compared to all control conditions (Figure 5A). CD107a, a marker of NK cell activity, was also upregulated in RSV-infected NK cells (Supplementary Figure 4). In agreement with the results obtained for infection by RSV in the absence of antibodies, infection of neonatal NK cells by RSV-antibody complexes resulted in even more IFN-γ–expressing cells (Figure 5B). Similar results were obtained using adult NK cells (Figure 5C and 5D). In summary, both neonatal and adult NK cells are prone to produce IFN-γ upon RSV infection, and ADE of infection increases the total number of IFN-γ–positive cells by increasing the number of infected cells.

### RSV Infection of NK Cells Does Not Enhance Perforin Secretion

In addition to IFN-γ secretion, an important NK cell function is the secretion of granzymes and perforins to induce target cell death. Upon encountering a cytotoxicity trigger, for example, virus-infected or tumor cells, NK cells rapidly release preexisting granules containing both granzymes and perforins. Secretion of these granules results in a loss of intracellular perforin staining, which can be detected by flow cytometry [25]. This experimental approach allows for the discrimination between infected and uninfected cell responses in the same well, which would not be possible by measuring perforin released in the supernatant. We determined the percentage of perforin-negative cells as a measure for perforin secretion in 5 different NK cell populations: (1) mock-infected control cells without K562 target cells; (2) mock-infected control cells with target cells; RSV-exposed (3) infected and (4) uninfected cells with target cells; and (5) uninfected cells exposed to RSV in the presence of TMC with target cells.

**Figure 6.**
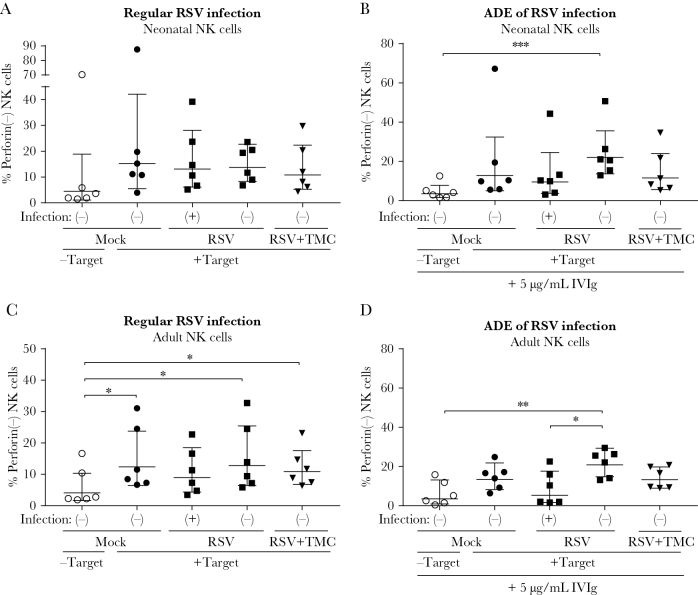
Respiratory syncytial virus (RSV) infection of natural killer (NK) cells does not enhance cytotoxicity. Neonatal (*A* and *B*) or adult (*C* and *D*) NK cells were infected with RSV-X, inoculated with RSV-X and TMC, or mock-infected. RSV-infected and uninfected populations are indicated with (+) and (-), respectively. At 20 hours postinfection, NK cells were incubated for 4 hours with brefeldin A in the absence or presence of K562 target cells and subsequently stained for intracellular perforin. *A* and *C*, Percentage of NK cells negative for intracellular perforin staining is depicted for control NK cells (with or without target cells), RSV(+) and RSV(-) cells within 1 RSV-inoculated well, and for NK cells inoculated with RSV and TMC. *B* and *D*, Same as *A* and *C* except for preincubation of the viral inoculum with 5 µg/mL intravenous immunoglobulin, resulting in antibody-dependent enhancement. All graphs depict geometric mean and standard deviation of n = 6 (neonatal) or n = 8 (adult) donors pooled from 2 (neonatal) or 4 (adult) independent experiments. Nonparametric Friedman test with Dunn multiple comparisons test was used for comparison between conditions (*P < .05, **P < .01, ***P < .01). Abbreviations: ADE, antibody-dependent enhancement; IVIg, intravenous immunoglobulin; NK, natural killer; RSV, respiratory syncytial virus.

Perforin secretion was slightly increased in all conditions upon the addition of target cells to neonatal NK cells (Figure 6A). Although RSV-infected NK cells are considerably more prone to produce IFN-γ than control cells (Figure 5A), this was not the case for perforin secretion. When neonatal NK cells were incubated with subneutralizing antibody concentrations (5 µg/mL IVIg), only the RSV-negative cells were significantly more likely to secrete perforin than unstimulated cells, possibly due to activation by neighboring opsonized RSV-infected NK cells (Figure 6B).

Since neonatal NK cells are described to be intrinsically less cytotoxic than adult NK cells [26, 27], we investigated whether the latter were more reactive in our cytotoxicity assay. Unlike neonatal cells, adult NK cells showed a significant increase in perforin secretion upon addition of target cells, supporting the reportedly low cytotoxic capability of neonatal NK cells (Figure 6C). Strikingly, only RSV-infected NK cells did not show significantly increased perforin secretion upon addition of target cells. ADE of NK cell infection enhanced this effect, resulting in a significant difference in perforin secretion between infected and uninfected NK cells in the same well (Figure 6D). These data suggest that, unlike what was seen for IFN-γ, the secretion of perforin is not enhanced and is possibly even reduced in RSV-infected adult NK cells.

## DISCUSSION

In the past, it was thought that epithelial cells were the only target of RSV [11, 28]. However, there is increasing evidence that RSV is also able to infect immune cells in humans [22, 23], and viral RNA has been detected in peripheral blood cells during acute infection [29]. In our experiments, infected NK cells did not release infectious viral particles, which is consistent with studies of RSV infection in other immune cells [24, 30, 31]. Infection of NK cells has been documented before for several unrelated viruses (including human immunodeficiency virus, vaccinia virus, human herpesvirus 6, and influenza virus), and only some of these establish productive infections [32–35].

Considering that NK cells, recruited to the lungs during infection in infants, likely encounter RSV bound to (subneutralizing levels of) virus-specific antibodies, it is probable that Fc gamma receptor (FcγR)–mediated antibody effector functions are activated. Recently, researchers found that antibodies with enhanced binding to CD16/FcγRIII determined disease severity in dengue infection [36]. Moreover, in vitro ADE of RSV infection has been shown before by several groups including our own [37–39]. In the current study, we show that in the presence of subneutralizing antibody concentrations, infection of NK cells can be enhanced up to 4-fold for adult NK cells and up to 6-fold for neonatal NK cells, resulting in a substantial proportion of infected NK cells in vitro. Further research is needed to determine the exact contribution of CD16/FcγRIII to ADE of RSV infection in NK cells.

We show that RSV infection of NK cells results in increased numbers of IFN-γ–producing cells. In mouse models of RSV infection, NK cells were shown to be the most important source of IFN-γ early after infection [4, 5]. However, the role of IFN-γ in RSV disease has been much debated. On the one hand, it has been suggested that IFN-γ is involved in acute lung injury, allergic airway disease, and airway obstruction in animal models [5, 40, 41] and appears to be associated with virus-induced wheezing in humans [42]. On the other hand, decreased IFN-γ levels were found in the blood and in the nasopharyngeal aspirates of mechanically ventilated infants compared to nonventilated infants [43, 44]. However, it is debatable whether measurements in the blood accurately reflect IFN-γ levels in the lungs during RSV infection. Also, in a more recent study, IFN-γ levels were found to be elevated during RSV bronchiolitis when measured by nasosorption, but not in nasopharyngeal aspirates, showing a clear effect of sampling technique [45]. Considering these findings, it seems that the detrimental effects of IFN-γ are an exacerbated outcome of an intrinsically beneficial role for this molecule in protection against RSV disease. We have shown that RSV infection of NK cells leads to more IFN-γ–producing cells, a potential way through which the virus disturbs the balanced NK cell response. In contrast to the increase in IFN-γ production in RSV-infected NK cells, we did not observe more perforin secretion upon RSV infection. Adult NK cells infected in the presence of RSV-antibody complexes even showed significantly lower perforin secretion than uninfected cells in the same well. This effect is likely only seen in adult NK cells because of the intrinsically lower capacity of neonatal NK cells to elicit a cytotoxic response. This is also evidenced by the almost 10-fold lower MFI for perforin in neonatal compared to adult NK cells (data not shown). Overall, we only observed minor differences between neonatal and adult NK cells. This was an unexpected finding, as earlier studies show more RSV infection in neonatal compared to adult monocytes [46] and severely decreased cytotoxic responses by neonatal NK cells [26, 27]. Our experiments were performed with the addition of small amounts (5 ng/mL) of IL-15. This has been shown to increase the functionality of neonatal NK cells [27] and possibly explains the limited differences between adult and neonatal NK cells in our study. Moreover, high variability has been observed between cord blood donors in earlier studies [47] with some donors exhibiting similar or even higher cytotoxicity levels than adult donors.

Of note, in our experiments, CD107a expression correlated with IFN-γ production but not with perforin secretion. This suggests that CD107a is not merely a secretion marker of perforin, but acts as a more general marker for NK cell activity.

Increased IFN-γ production together with a possible inhibition of perforin secretion suggests a shift toward a proinflammatory rather than a cytotoxic NK cell state. In addition, the phenotypic characteristics of RSV-infected NK cells suggest skewing toward a less cytotoxic and more inhibitory phenotype. This may impair the cytolytic function of these cells, as has been shown for other viruses [35, 48]. Based on previous reports and the data described in this article, we propose that the proinflammatory response of RSV-infected NK cells may contribute to the development of RSV-mediated severe disease. Notably, the formalin-inactivated RSV vaccine that caused enhanced disease upon natural infection induced primarily nonneutralizing antibodies [49]. Our data support the idea that these antibodies may have contributed to enhanced inflammation [50].

To conclude, we have shown that RSV-infected NK cells are more prone to produce IFN-γ than uninfected cells, whereas the cytotoxic response is not increased. This combination may contribute to RSV immunopathology in vivo, but more research is needed to support this hypothesis. Moreover, we show that Fc-mediated effector functions such as ADE of infection can have a profound effect on the immune response and a potential role in the development of disease. Our findings contribute to the understanding of the potential mechanisms responsible for the development of severe RSV disease and highlight the critical need to further characterize the role of NK cells and RSV-antibody complexes therein, as this will assist in the development of safe and effective RSV vaccines.

## Supplementary Data

Supplementary materials are available at *The Journal of Infectious Diseases* online. Consisting of data provided by the authors to benefit the reader, the posted materials are not copyedited and are the sole responsibility of the authors, so questions or comments should be addressed to the corresponding author.

Supplementary Figure 1

Supplementary Figure 2

Supplementary Figure 3

Supplementary Figure 4

Supplementary Figure captions

## Notes

Presented in part: Royal Dutch Society for Microbiology Scientific Spring Meeting, Papendal, Netherlands, 27 March 2018; American Society for Virology Annual Meeting, College Park, Maryland, 16 July 2018; and European Congress of Immunology, Amsterdam, Netherlands, 3 September 2018.

***Acknowledgments.*** We thank T. Guichelaar (RIVM) and J. de Wit (RIVM) for helpful discussions, and A. Nicolaie (RIVM) for help with the statistical analyses.

***Financial support.*** E. v. E., O. W., and P. v. K. received funding from the Dutch Ministry of Health, Welfare and Sport Strategic Program (RIVM S/112008); G. F. received funding from the Virgo consortium (FES0908) and the Netherlands Genomics Initiative (050-060-452).

***Potential conflicts of interest.*** All authors: No reported conflicts of interest. All authors have submitted the ICMJE Form for Disclosure of Potential Conflicts of Interest. Conflicts that the editors consider relevant to the content of the manuscript have been disclosed.
