# Supplementary figures and images for: Respiratory Syncytial Virus Infects Primary Neonatal and Adult Natural Killer Cells and Affects Their Antiviral Effector Function
Source: J Infect Dis. 2018 Sep 25;219(5):723–33. doi: 10.1093/infdis/jiy566 (PMC6376914; doi:10.1093/infdis/jiy566)

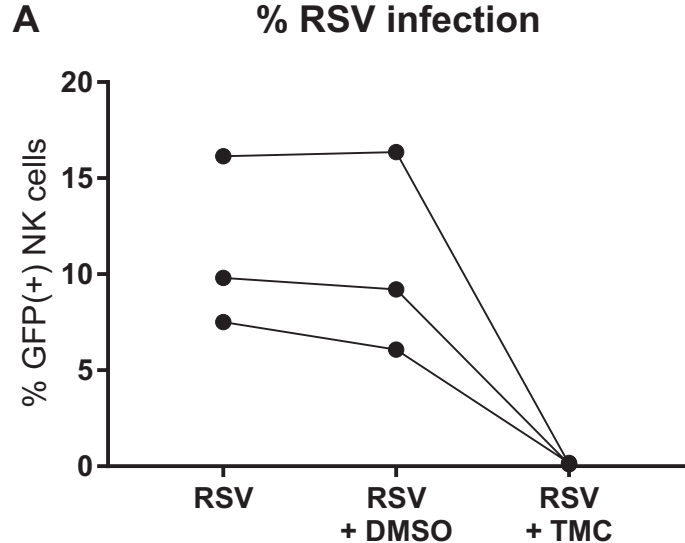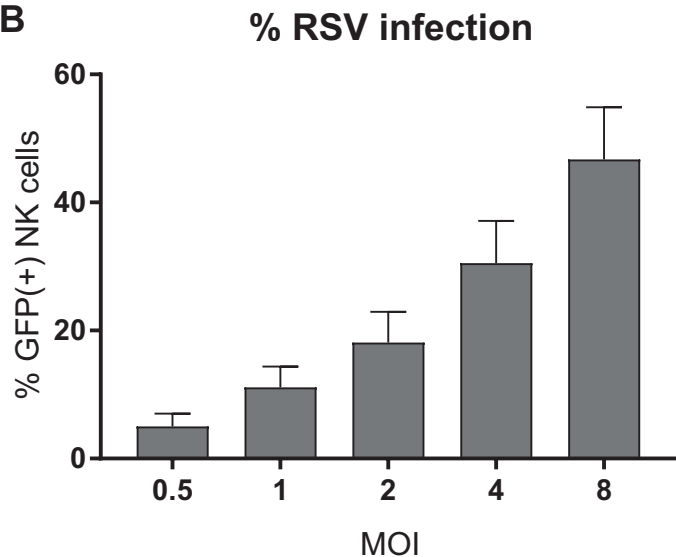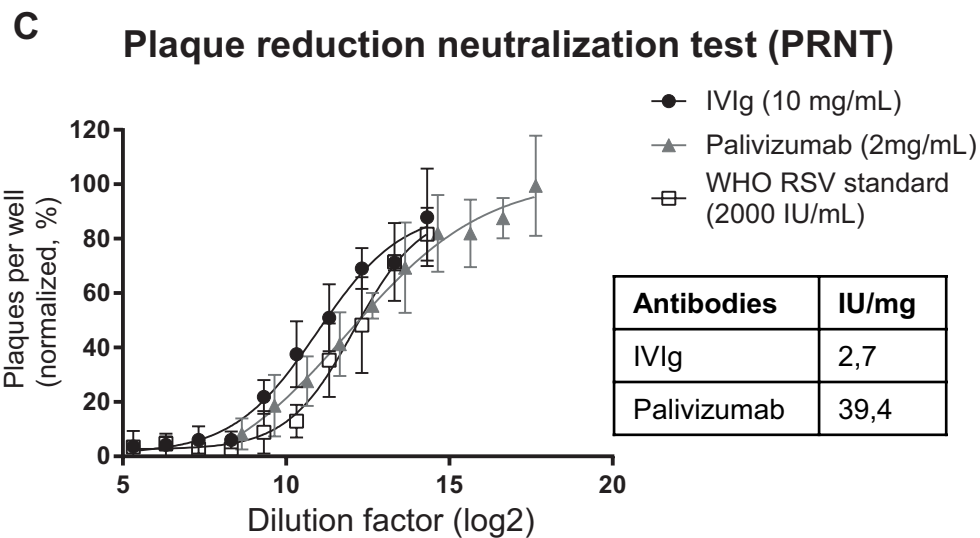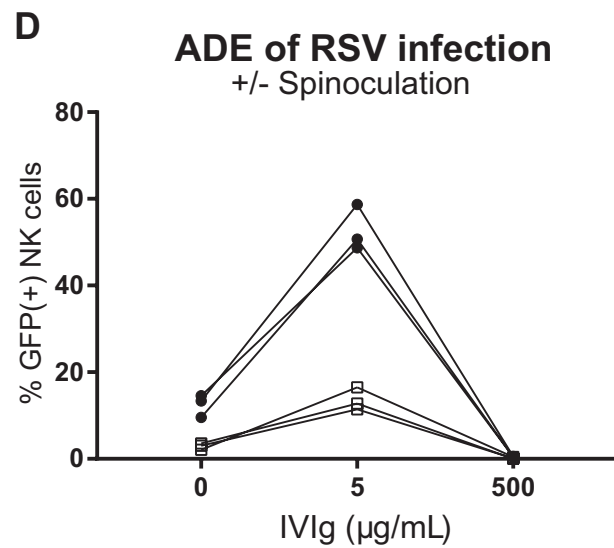

Supplement: Supplementary Figure 1 [file jiy566_suppl_supplementary_figure_1.pdf]

## A. Activating receptors

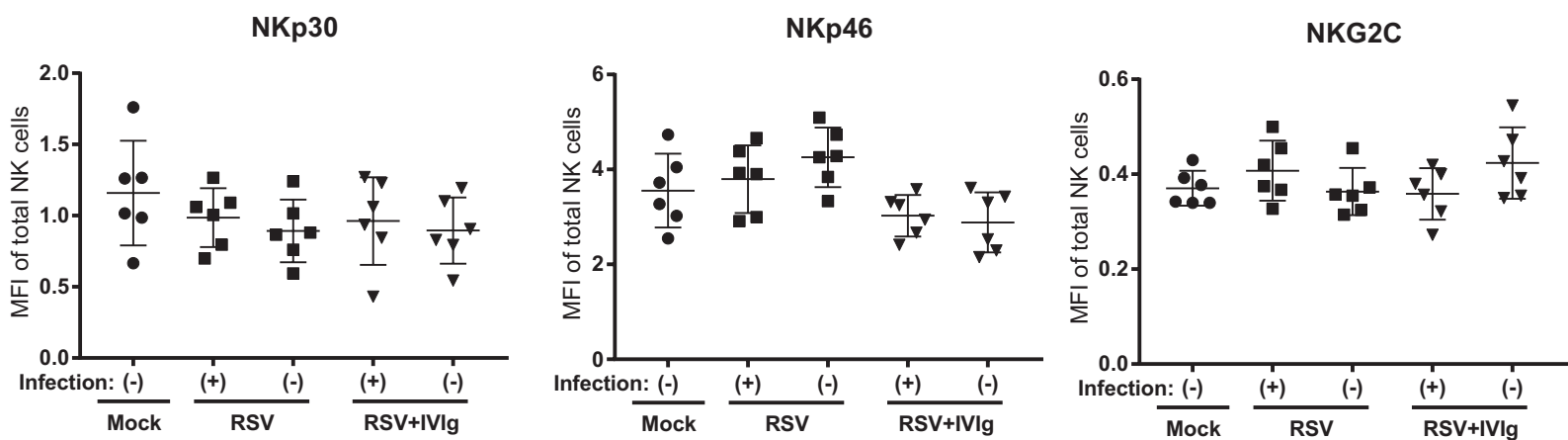

## B. KIRs

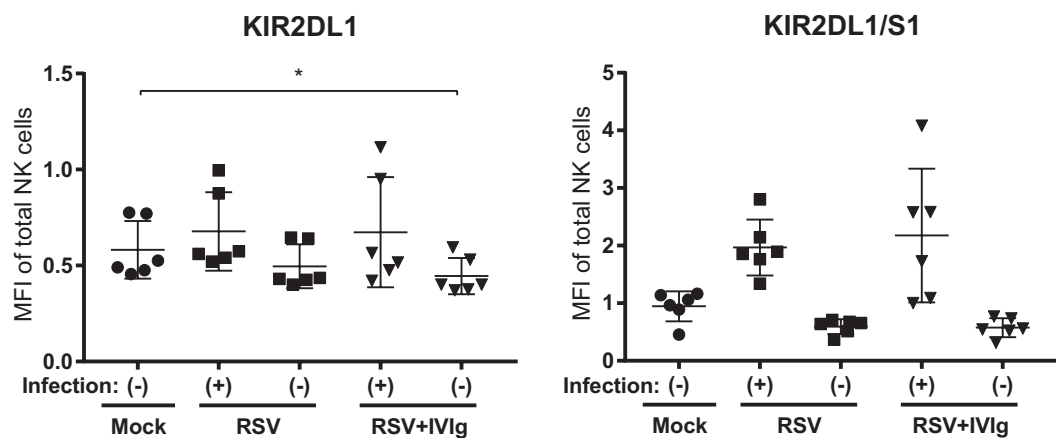

## C. Inhibitory receptors

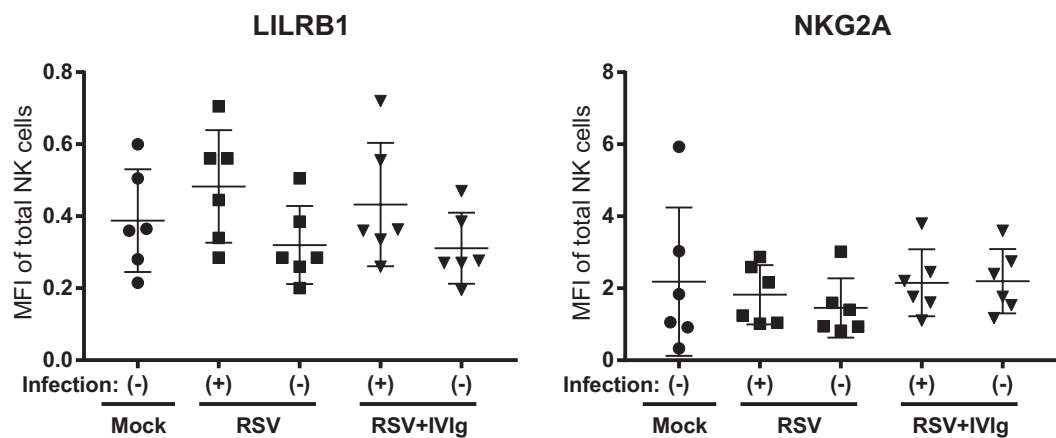

## D. Dual-function receptors

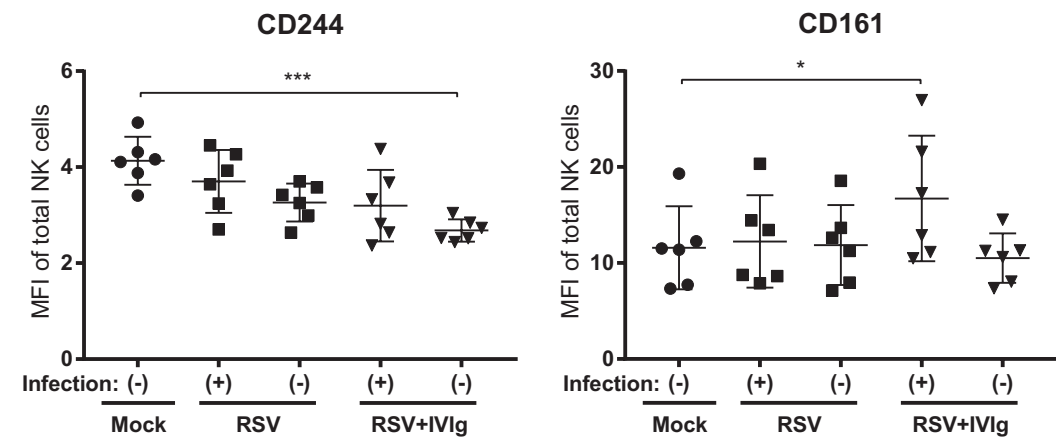

Supplement: Supplementary Figure 2 [file jiy566_suppl_supplementary_figure_2.pdf]

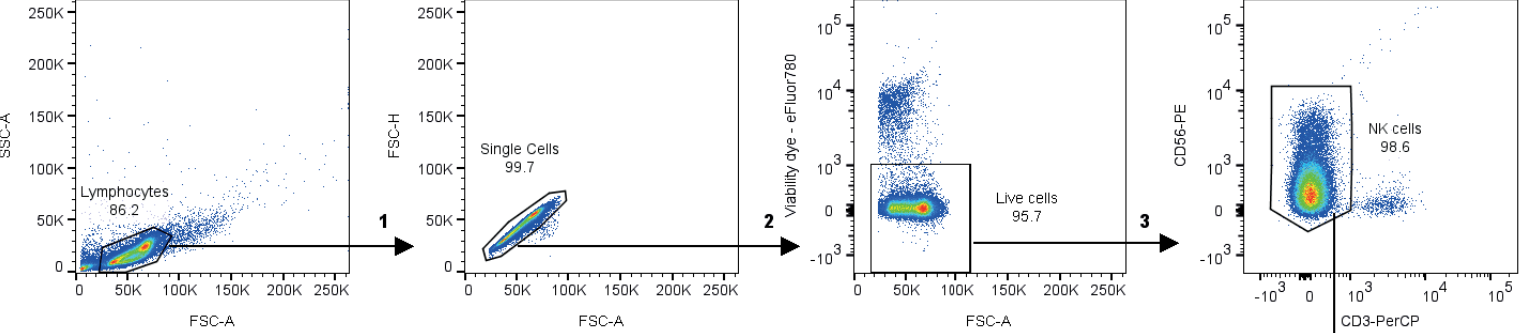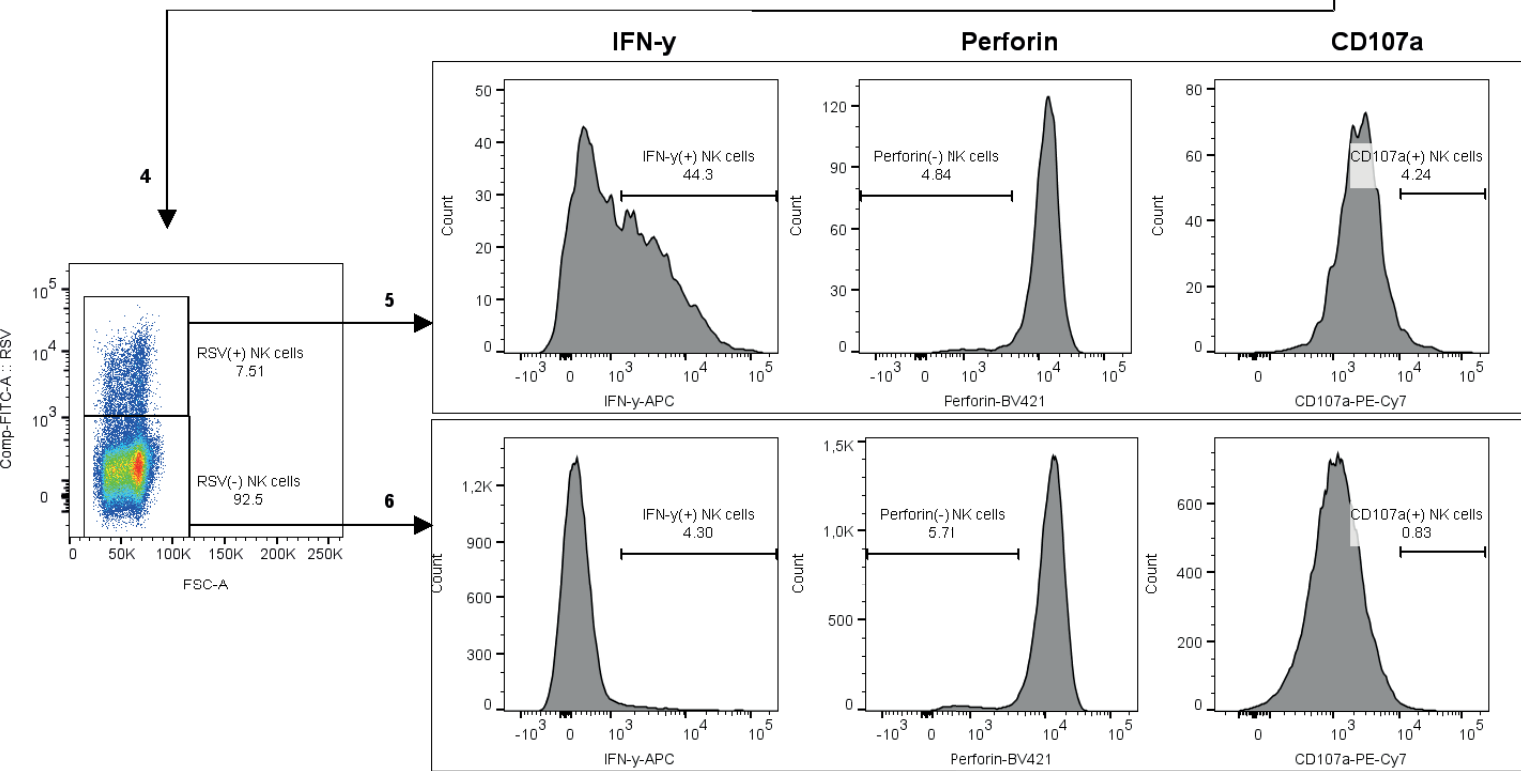

Supplement: Supplementary Figure 3 [file jiy566_suppl_supplementary_figure_3.pdf]

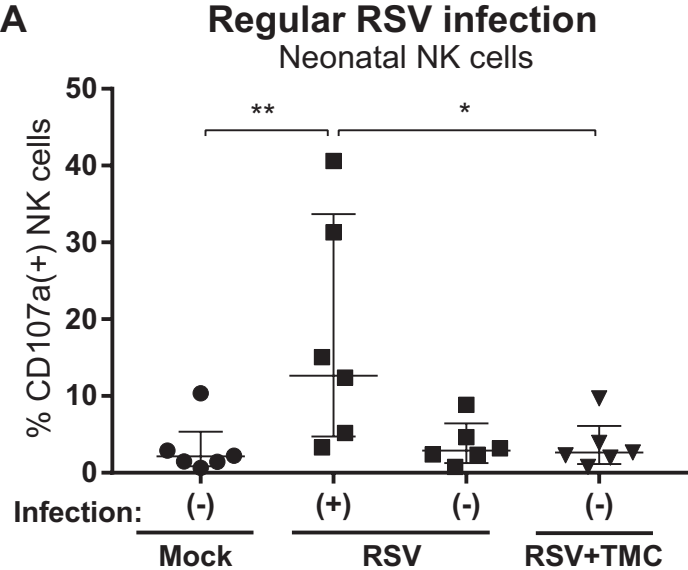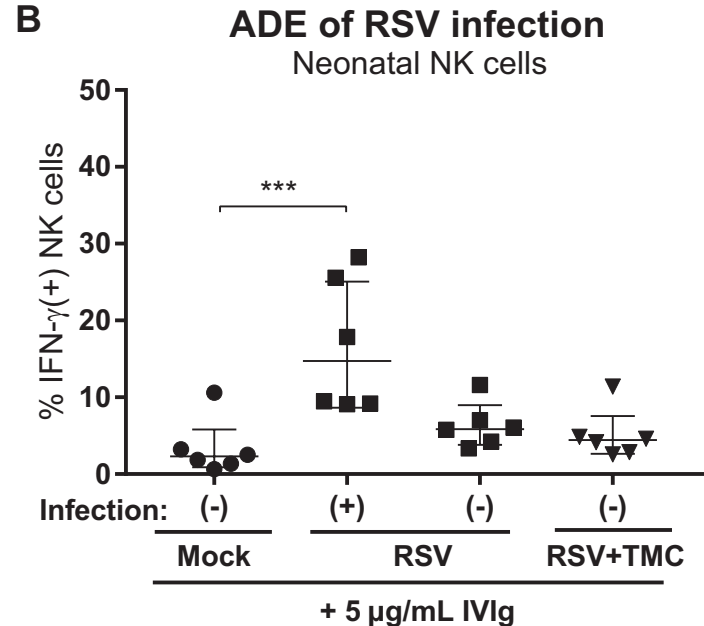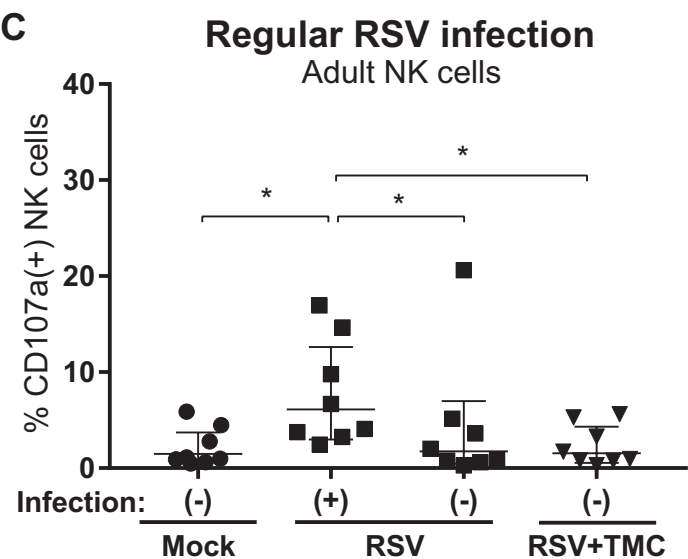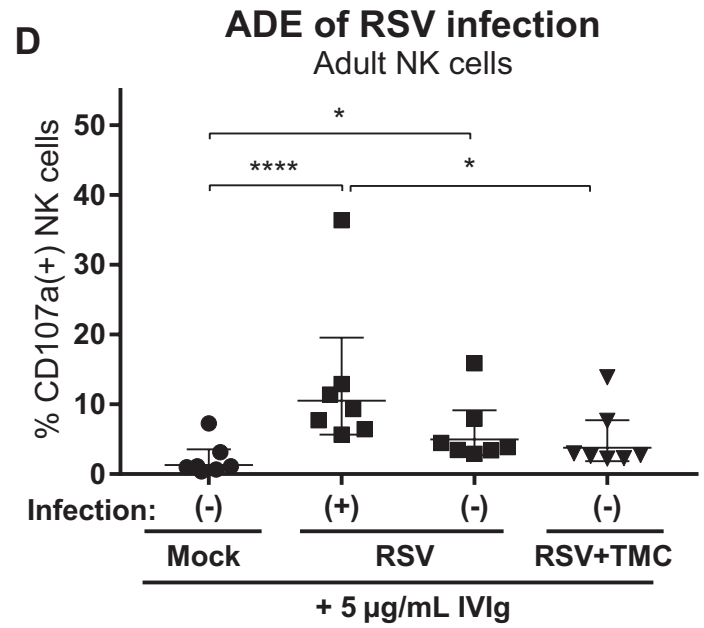

Supplement: Supplementary Figure 4 [file jiy566_suppl_supplementary_figure_4.pdf]
