# Supplementary material for: Respiratory Syncytial Virus Infects Primary Neonatal and Adult Natural Killer Cells and Affects Their Antiviral Effector Function
Source: J Infect Dis. 2018 Sep 25;219(5):723–33. doi: 10.1093/infdis/jiy566 (PMC6376914; doi:10.1093/infdis/jiy566)
Supplement: Supplementary Figure captions [file jiy566_suppl_supplementary_figure_captions.docx]

# Supplementary Figure captions

**Fig S1. RSV infection of NK cells and antibody-dependent enhancement** – (A) Adult primary NK cells were inoculated with RSV-X-GFP7 in the absence or presence of a fusion inhibitor (TMC) or vehicle control (DMSO). Infection was measured by flow cytometry at 20 h.p.i. Each set of paired data points represents an individual donor (n=3) tested in one experiment. (B) NK cells were inoculated with increasing MOIs of RSV-X-GFP7 as indicated. GFP expression was measured at 20 h.p.i. by flow cytometry. Graph depicts geometric mean and SD of n=3 individual donors from one experiment. (C) Neutralization curves for serially diluted Intravenous Immunoglobulin (IVIg), Palivizumab or the WHO RSV reference serum. Graph depicts geometric mean and SD from triplicates in one experiment. (D) Adult primary NK cells were infected with RSV-X-GFP7 by spinoculation for 1 h at 700x*g* at 20°C, followed by 1 h incubation at 37°C (closed circles), or by 2 h incubation at 37°C, without spinoculation (open squares). For both conditions, each set of paired data points represents an individual donor (n=3) tested in one experiment.

**Fig S2. Phenotypic characterization of RSV-infected NK cells** – (A-G) Adult primary NK cells were inoculated with RSV or RSV-antibody complexes (5 µg/mL IVIg) and stained with 3 different antibody panels for flow cytometric analysis at 20 h.p.i. Geometric mean of fluorescence intensity (MFI) is depicted for the markers not included in Figure 3: (A) activating receptors (NKp30, NKp46, and NKG2C), (B) KIRs (KIR2DL1 and KIR2DL1/S1), (C) Inhibitory receptors (LILRB1 and NKG2A), and (D) undefined (dual-function) receptors (CD244 and CD161). Graphs depict the geometric mean and SD of n=6 donors pooled from two independent experiments. Non-parametric Friedman test with Dunn’s multiple comparison test was used for comparison between multiple conditions (*P<0.05, ***P<0.001).

**Fig S3. Gating strategy for NK cell activity assays** – Gating strategy for the NK cell activity assays, measuring IFN-γ, perforin, and CD107a. Dot plots and histograms are depicted for one representative adult NK cell donor.

**Fig S4. RSV infection of NK cells induces CD107a expression** – Neonatal (A-B) or adult (C-D) NK cells were infected with RSV, inoculated with RSV and TMC, or mock-infected. RSV-infected and uninfected populations are indicated with (+) and (-), respectively. At 20 h.p.i., NK cells were incubated for 4 h with Brefeldin A and CD107a staining. (A/C) Percentage NK cells positive for CD107a depicted for control NK cells, RSV(+) and RSV(-) cells within one RSV-inoculated well and for NK cells inoculated with RSV and TMC. (B/D) Same as (A/C) except for pre-incubation of the viral inoculum with 5 µg/mL IVIg, resulting in ADE. All graphs depict geometric mean and SD of n=6 (neonatal) or n=8 (adult) donors pooled from two (neonatal) or four (adult) independent experiments. Non-parametric Friedman test with Dunn’s multiple comparison test was used for comparison between conditions (*P<0.05, **P<0.01, ***P<0.001, ****P<0.0001).
